# Investigating Properties of Palmitoylethanolamide in Physiology and Disease: Far Beyond an Anti-Inflammatory Shield

**DOI:** 10.3390/diseases14020052

**Published:** 2026-01-31

**Authors:** Chiara Veredice, Ida Turrini, Helena Pelanda, Ilaria Contaldo, Donato Rigante

**Affiliations:** 1 Pediatric Neurology Unit, Department of Woman and Child Health and Public Health, Fondazione Policlinico Universitario A. Gemelli IRCCS, 00168 Rome, Italy; chiara.veredice@policlinicogemelli.it (C.V.); ida.turrini@policlinicogemelli.it (I.T.); ilaria.contaldo@policlinicogemelli.it (I.C.); 2 Department of Life Sciences and Public Health, Fondazione Policlinico Universitario A. Gemelli IRCCS, 00168 Rome, Italy; helena.pelanda01@icatt.it; 3 Università Cattolica Sacro Cuore, 00168 Rome, Italy

**Keywords:** palmitoylethanolamide, *N*-acylethanolamide, chronic pain, neurogenic inflammation, neuroinflammation, endocannabinoidome, personalized medicine, bio-innovative therapies

## Abstract

Palmitoylethanolamide (PEA) among *N*-acylethanolamides displays a noteworthy impact on different inflammatory conditions and promises to become a valuable anti-inflammatory tool that does not interfere with the cyclooxygenase pathway. Mounting evidence confirms the multi-dimensional PEA-mediated crosstalk between microglia and mast cells, which would open new therapeutic opportunities targeting a neuroimmune axis and influencing both health and disease. In particular, PEA acts as a preserver of cellular homeostasis by regulating microglia cell activity and inhibiting mast cell activation in the central nervous system. The improved bioavailability and efficacy of ultramicronized formulations of PEA reflect its ultimate usefulness for different clinical applications, including significantly relieving inflammation but also reducing the pro-inflammatory burden of complex patients with either neuropathies or non-neurologic afflictions. This review aims to comprehensively delineate the therapeutic potential of PEA beyond its mere indication for acute inflammation and to highlight PEA activity as a broad-spectrum pan-tissue protective agent through the results of different preclinical and also some clinical studies. Much more remains to be learned about further PEA mechanisms of action that regulate neuroinflammation, and additional studies will have to investigate the exact role of microglia and mast cells in inflammatory diseases.

## 1. Introduction

Nutraceutics are functional components of food with positive effects on health which may fight inflammation, as evidenced by many studies evaluating chronic pain and oxidative burden in different protean conditions. Specifically, the term “nutraceutic” is a neologism coined three decades ago from the words ‘nutrient’ and ‘pharmaceutical’ to juxtapose concepts defining a food product that favors the human body’s physiological processes [1]. Such elements displaying positive sequels for well-being may also be active in both the prevention and treatment of diseases, without having calorific and nutritive power, and their list is expanding from resveratrol and isoflavones to omega-3 fatty acids. However, data related to their intake in different age groups have been provided by nationwide nutrition surveys in a fragmented fashion [2]. Peculiar changes of the gut microbiome caused by Western dietary patterns have been related to altered consumption of different nutrients, and there is a fervid interest in non-drug treatments deriving from food that can help improve health since childhood. Indeed, many bioactive substances might act as important modulators of wellness through their effects on metabolism and immunity.

In particular, *N*-acylethanolamides are endocannabinoid-like fatty acid derivatives present in some alimentary products that have been proven to modify bacterial growth and affect many physiologic functions through an indirect modulation of the gut microbiome [3]. The presence of *N*-acylethanolamides and their precursors in various tissues and their overall pharmacological properties suggest their role as autocrine regulators of cell life. Furthermore, the existence of *N*-acylethanolamides in the mammalian brain has been recognized for decades, while—specifically among *N*-acylethanolamides—palmitoylethanolamide (PEA or *N*-acylethanolamine) has been found to be particularly abundant in the central nervous system, being largely produced by both neurons and glial cells to protect brain areas in neurodegenerative diseases such as Alzheimer’s and Parkinson’s disease, but also in demyelinating diseases and traumatic brain injuries. For instance, Franklin et al. found that levels of PEA increase after focal ischemia in a mouse cerebral cortex, leading to hypermobility of microglial cells [4]. PEA is physiologically synthesized ’’on demand’’ within the lipid bilayer but is present as a pro-homeostatic factor in almost all tissues of the body, with levels increasing in response to cellular damage [5,6,7]. At minimal non-therapeutic concentrations, PEA is naturally present in various food sources, including soy, chia, egg yolk, apples, lentils, black-eyed peas, potatoes, roasted coffee, and human milk, but numerous nutraceuticals as well as dietary supplements marketed in various countries have had PEA added to them due to its considerable anti-inflammatory power [8,9].

## 2. Key Information About the Molecular Effects of Palmitoylethanolamide

PEA works as an endogenous pleiotropic mediator and has pivotal functions in the autacoid plot to contrast local injuries and favor the maintenance of tissue homeostasis by regulating immune cells, microglia, and non-neuronal cells, specifically stabilizing mast cells and preventing their degranulation [10]. The anti-inflammatory activity of PEA directly bypasses the prostaglandin biosynthesis pathway, which is targeted by the conventional nonsteroidal anti-inflammatory drugs, and from within the central nervous system involves mast cells and microglia [11,12]. The key downstream interacting molecule of this regulatory function for PEA is the nuclear receptor peroxisome proliferator-activated receptor-α (PPAR-α), a ubiquitous transcription factor that is also a lipid sensor that interacts with many metabolic pathways and finally inhibits both the nuclear factor kappa-light-chain-enhancer of the activated B-cell (NF-κB) and the mitogen-activated protein kinase (MAPK) signaling [13]. In addition to PPAR-α, PEA can also activate different receptors and inhibit some ion channels involved in the response to neuronal firing, e.g., vanilloid receptor and K+ channels; furthermore, through activation of PPAR-α, PEA may stimulate neurosteroid synthesis, thereby amplifying several biological responses mediated by gamma aminobutyric acid A receptors [14]. This specific activity may mimic the activity of endocannabinoids and also enhance the production of both anandamide (AEA) and oleoylethanolamide (OEA). However, though structurally similar to AEA and OEA, PEA has no selective interaction with cannabinoid receptors [15,16].

Several non-genomic and delayed genomic mechanisms of action have been identified for PEA: acting on PPAR-α, which tunes the expression of negative regulators of inflammation such as IκB-α, repressing the release of tumor necrosis factor (TNF)-α, and limiting the recruitment of immune cells within injured sites. LoVerme et al. found that PPAR-α agonists exert a rapid anti-nociceptive effect in animal models of neuropathic pain, similar to the gabapentin effect but without development of tolerance. Overall, the integration of different modes of action allows PEA to exert a prolonged command in neuron signaling, either on membrane excitability or activation of inflammatory cells [17]. A multifaceted pharmacological profile beyond the well-known regulatory role in inflammation has been recognized for PEA, whose metabolic and immunologic activities crosstalk with energy balance, fatty acid oxidation, and gene expression within the macrophages and lymphocytes of both humans and animals [18,19,20].

The multi-target effects of PEA may also influence neurogenic inflammation, occurring as a core feature of migraine pathophysiology, and an important clinical trial assessing the management of headaches has emphasized the efficacy of ultramicronized PEA (um-PEA) for the prophylaxis of migraine in children [21]. In particular, through stabilization and direct inhibition of mast cell degranulation, um-PEA may suppress the release of pro-inflammatory mediators active in the headache cycle, reducing both the frequency and intensity of attacks. Importantly, Impellizzeri et al. evaluated the oral anti-inflammatory efficacy of micronized versus non-micronized PEA formulations in the paw of a murine model of inflammation induced by intra-plantar injection of carrageenan, finding that paw edema and thermal hyperalgesia were most reduced by the micronized PEA formulation [22]. In addition, um-PEA has also been shown to be clinically effective at decreasing systemic inflammation from relapsing-remitting multiple sclerosis, lowering the circulating levels of interferon (IFN)-gamma and interleukin (IL)-17, and even improving the cognitive component of patients’ quality of life [23].

The analgesic and anti-inflammatory activity of PEA has been investigated in a wide variety of inflammatory conditions, though mostly with preclinical studies, finding that PEA and its derivative adelmidrol can actively shield against the oxidative microenvironment through a PPAR-α-mediated activation of the Nrf2 (nuclear factor erythroid 2-related factor 2) signaling pathway. Fusco et al. assessed both inflammation and oxidative stress development and the potential efficacy of a combined treatment with PEA and rutin in a mouse model of vascular injury provoked by the ligature of the left carotid artery for 14 days: the authors found that vascular morphology was improved by PEA/rutin, while the release of adhesion molecules as well as pro-inflammatory cytokines like IL-1 β, IL-6, and TNF-α was reduced and oxidative stress was mitigated [24]. More recently, interest in PEA has steadily increased, and many researchers have examined the potential for a translation of PEA preclinical studies into clinical practice in the specific field of inflammatory diseases involving the central nervous system and in personalized medicine strategies [25].

## 3. The Role of Mast Cells in the Microglia Activation

**Figure 1:**
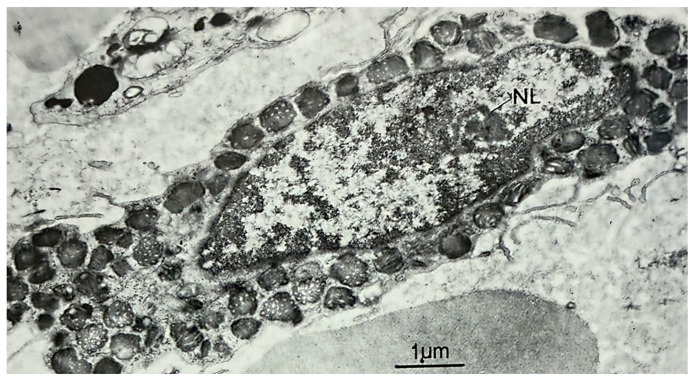
Mast cell has a single oblong nucleus with a small nucleolus (NL) combined with highly characteristic cytoplasmic granules having a scroll-like ultrastructure visible on electron microscopy (adapted from Ref. [37]).

Some molecular studies have disclosed multiple PEA capacities, from anti-inflammatory to neuroprotective, related to the specific involvement of mast cells, which have been shown to play a pivotal role in inflammatory pathways. These cells are etiologically implicated in many pathologies, including type I hypersensitivity reactions, mastocytosis, and urticaria, but they also work as critical sentinels that detect threats like pathogens or external triggers of different nature and start inflammation by releasing variably potent chemicals to widen blood vessels, attract other immune cells, and fight the threat itself, while also regulating wound healing, angiogenesis, and nerve functions [26]. Figure 1 shows an electron microscopic image of a mast cell, in which the prominence of numerous cytoplasmic granules can be clearly seen. Increasing evidence suggests that the crosstalk between mast cells and glia has a relevant role in the development of neuroinflammation, exacerbating the acute inflammatory response, accelerating neurodegenerative pathologies, and promoting the perception of pain [27]. In particular, mast cells can produce a potentially lethal cocktail of compounds capable of damaging neurons and glial cells. Despite being localized in loose connective tissue throughout the body (but predominantly in the skin, airways, gastrointestinal tube, urinary and reproductive tracts), there are brain-resident mast cells secreting an array of neuroregulatory mediators to actively participate in the protection of the central nervous system. After entering the brain—in the earlier phases of development—mast cells become capable of antigen presentation and phagocytosis [28]. Herein, they act as first “responders” to potential local injuries, interacting with neurons and microglia, and release proteases and reactive oxygen species: this process disrupts blood–brain barrier permeability, fueling inflammatory responses within the brain and finally facilitating tissue repair. The number of brain mast cells can fluctuate under stress, as shown by Nautiyal et al. who used genetically and pharmacologically modified mice with mast cell deficiency to evaluate their propensity to develop anxiety-like behaviors [29]. Indeed, the mast cell–neuron axis is a crucial system bringing about the release of pro-inflammatory mediators such as IL-4, IL-13, TNF-α, and tryptase: IL-4 and IL-13 promote the differentiation of T helper 2 cells, while TNF-α and tryptase promote dendritic cell maturation and enhance IL-12 production, thereby facilitating Th1 polarization [30]. Anti-TNF therapy may successfully alleviate severe systemic inflammation occurring at the neurologic level in patients with autoinflammatory disorders, controlling the severity of disease flares and preventing amyloidosis specifically in TNFR-associated periodic fever syndrome, an autosomal dominantly-inherited disease linked to chromosome 12p13 and, more specifically, to mutations within the TNFR superfamily member 1A gene [31,32,33]. Moreover, mast cell-derived IL-4 also activates the STAT6 signaling pathway in dendritic cells, upregulating the chemokine ligands (CCL)17 and CCL22, which recruit Th2 cells and promote a Th2-skewed immune microenvironment within the inflamed tissue: this contributes to modulating the local immune response that prompts microglia functional changes and their switch to a pro-inflammatory phenotype [34,35]. Furthermore, microglia-mediated inflammation has been proposed to compromise brain aging, and the repertoire of molecules seen in neuroinflammation has been dragged into many disorders of the central nervous system, from the simplest neuropathic pain and epilepsy to even neurodegenerative diseases [36].

## 4. A Specific Enterprise of Palmitoylethanolamide in Neuroinflammation

The administration of PEA has been used to increase the magnitude of innate immunity responses through the activation of nonspecific pathways that work against viral and bacterial pathogens and the regulation of mast cell degranulation, as revealed by the experiments conducted on patients with severe acute respiratory syndrome coronavirus-2 infection [38]. The number of clinical studies evaluating PEA effects in neuroinflammation is rather limited, though a plethora of pro-inflammatory cytokines, eicosanoids, and other immune neurotoxins have been found in the cerebrospinal fluid of patients with neurodegenerative disorders. The anti-inflammatory actions of PEA, leading to a reduction in peripheral and central sensitization, are mediated by neuronal and non-neuronal cells, which comprise microglia and astrocytes, as well as peripheral and central mast cells. Moreover, it has been proven that PEA reduces neuron loss and that—when locally administered at different doses—it also reduces in a concentration-dependent manner the expression and release of nerve growth factor in an animal model of chronic carrageenan-induced inflammation, modulating mast cell degranulation in proximity to nerves and finally decreasing allodynia and mechanical pain [39].

The lipidic nature of PEA and its large size at its native state might limit its solubility and bioavailability when given orally, but micronized formulations of PEA enhance its rate of dissolution; in fact, both micronized and nano-sized formulations have an improved absorption profile, as shown by Petrosino et al. [40]. Additionally, a double-blind randomized placebo-controlled study, testing the effect of um-PEA added to IFN-β1a in the treatment of 29 patients with relapsing-remitting multiple sclerosis, found that those treated with um-PEA perceived an improvement in IFN-β1a-related side effects and a significant improvement in quality of life, combined with a substantial reduction of IFN-γ, TNF-α, and IL-17 compared to patients treated with a placebo [41].

PEA has shown significant efficacy in the treatment of chronic neuropathic pain: Lang-Illievich et al. undertook a meta-analysis to identify double-blind randomized controlled trials comparing PEA (at dosages ranging from 300 to 1200 mg per day for 8–12 weeks) versus placebo to assess its analgesic efficacy in patients with neurological diseases, gynecological pain, musculoskeletal disorders, and irritable bowel syndrome; PEA was shown to increase quality of life and improve sleep quality while reducing overall patients’ symptom severity [42]. Furthermore, starting from the assumption that recurrent migraine attacks may depend on neurogenic inflammation and subsequent output of pro-inflammatory cytokines, De Icco et al. studied 24 subjects with migraine who were administered nitroglycerin to experimentally induce a clinical attack and found that plasma PEA was significantly increased 120 min after nitroglycerin exposure [43]. Hernàndez performed an open-label experimental study involving 25 adult patients with episodic migraine (with and without aura) in whom treatment with PEA, taken every 12 h for 3 months, reduced the monthly attack frequency as well as the attack intensity and quality of sleep [44]. Chirchiglia et al. published the results of a single-blind study to evaluate both the safety and efficacy of umPEA (at a dose of 1.200 mg/day) given for 90 days in 20 adult patients suffering from migraine with aura, in whom they found statistically significant pain relief after 60 days of treatment [45]. Petrosino et al. wanted to text PEA levels in some rat brain areas involved in nociception, i.e., the dorsal raphe and rostral ventral medulla as well as in the spinal cord, following sciatic nerve constriction: PEA levels were found to have significantly decreased in the spinal cord three days after injury, and in the dorsal raphe and rostral ventral medulla seven days after injury [46].

A complex blend of genetic and environmental factors—like advanced parental age, prenatal exposure to certain pollutants or medications and birth complications—may predispose to the onset of autism-spectrum disorder (ASD). There is some evidence that reduced levels of endocannabinoidome mediators like AEA and OEA may specifically drive ASD. Campanale et al. suggested that PEA helped manage ASD-related symptoms and that mucin-degrading genera, like *Akkermansia* and *Ruminococcus*, which modulate the levels of endocannabinoidome mediators, may also give some therapeutic advantage to children with ASD [47]. Theoharides et al. studied the perinatal non-allergic activation of mast cells by infections or stress-related triggers, finding that the release of neurotoxic molecules contributed to in utero brain inflammation, which could induce preterm labor and even dysfunctional activity in particular brain areas with future potential risk of developing ASD [48].

Ischemic stroke is strongly correlated with neurogenic inflammation involving neural cells, such as microglia and astrocytes, as well as peripheral immune cells: in particular, a cascade of inflammatory signaling pathways follows PPAR-α involvement and promotes a macrophage polarization shift from the pro-inflammatory M1 to the anti-inflammatory M2 phenotype, crucial for clearing debris, promoting neuroplasticity, and facilitating functional recovery [49]. Brain histological damage has been significantly prevented by PEA administration in an animal model of stroke induced by middle cerebral artery occlusion, leading to decreased NF-κB expression and neuronal apoptosis [50]. A similar neuroprotective effect through administration of um-PEA combined with luteolin was demonstrated in 60 patients affected by acute ischemic stroke and undergoing thrombolysis [51]. Various experimental studies have shown that PEA may counteract neuroinflammation and neuronal damage in Alzheimer’s disease and that PEA attenuates the tendency to neuronal atrophy and inflammatory cytokine production [52]. In fact, Facchinetti et al. tested the potentially beneficial effects of PEA combined with luteolin in an in vitro model of Alzheimer’s disease related to Aβ_1-42_ toxicity by triggering astrocyte reactivity and inflammation. The authors showed that these two drugs counteracted the Aβ_1-42_-induced inflammation [53].

Significant evidence is now being accumulated to highlight that microglial cells play a role in the degeneration of dopaminergic neurons in animal models of Parkinson’s disease, and that oxidative stress response by microglial cells is mediated through activation of the extracellular signal-regulated kinase signaling pathway by phosphorylation and translocation of both p47(phox) and p67(phox) cytosolic subunits [54]. In addition, PEA might prevent the loss of dopaminergic neurons, as Brotini et al. revealed after administering um-PEA (600 mg) as an adjuvant treatment in 30 Parkinson patients, routinely on levodopa therapy, who showed an improvement in their motor and non-motor symptoms like camptocormia [55].

Microglia cells also tend to exhibit an inflammatory pattern in Huntington’s disease, a neurodegenerative genetic disorder caused by a CAG repeat expansion in the huntingtin gene: specifically, striatal levels of endocannabinoids and PEA have been found to be significantly low in a transgenic mouse model of Huntington’s disease [56]. PEA can also modulate inflammation via PPAR-α activation in non-neuronal cells, improving the pain in patients with fibromyalgia, who have shown higher success rates if treated with PEA compared with duloxetine or pregabalin [57]. A report by Clemente observed the positive effect of PEA in a case of sporadic amyotrophic lateral sclerosis, a disease caused by mutations in the *TARDBP* gene, which involves the TAR DNA-binding protein-43, usually leading to motor neuron degeneration and frontotemporal dementia [58]. Finally, the retino-protective effects of PEA were studied by Ye et al. in a mouse model of oxygen-induced retinopathy, proving that PEA significantly reduces retinal gliosis via PPAR-α involvement [59].

There are different ongoing pilot studies and clinical trials assessing PEA in the areas of chronic neuropathic pain as well as in fibromyalgia, knee osteoarthritis, heat-induced hyperalgesia, bipolar depression, Tourette syndrome, diabetic neuropathy, chronic prostatitis, endometriosis, menstrual pain, glaucoma, eczema and coronavirus disease 2019: Table 1 lists the currently registered trials for patients with chronic pain syndromes, post-surgical pain, irritable bowel syndrome, acute stroke, mood disorders, and functional dyspepsia.

## 5. An Enlarging Spectrum of Activity for Palmitoylethanolamide in Human Diseases

PEA has been extensively studied for its substantial pain-reducing effects, showing both optimal tolerability and a good safety profile in humans. However, the number of trials and studies exploring PEA bioactivity in non-neurologic conditions is limited: the main suggestions of the available research are that PEA may give therapeutic benefits in various vascular, pulmonary, gastrointestinal, metabolic, hepatic, renal, rheumatological, and even oncological conditions [60]. The positive effects of PEA have been evaluated in some experimental models of vasculopathy and myocardial ischemia, revealing that treatment with um-PEA reduces myocardial tissue injury, neutrophil infiltration, expression of the adhesion molecules ICAM-1 and P-selectin, production of NF-κB, TNF-α, and IL-1β, formation of nitrotyrosine, and induction of apoptosis following a direct PPAR-α changeover [61].

Interdonato et al. studied a mouse model of acute lung injury (ALI) caused by intratracheal administration of lipopolysaccharide and tested the aerosolized administration of adelmidrol, a PEA analogue, given 1 and 6 h after lipopolysaccharide instillation: the authors found both reduced lung damage and reduced airway infiltration by inflammatory cells, hypothesizing that adelmidrol could downmodulate immune and specifically mast cells, decreasing markers of mast cell activation, i.e., chymase and tryptase [62]. Peritore et al. examined the effects of um-PEA administration in another mouse model of lipopolysaccharide-induced ALI, finding an improved lung picture in PEA-treated cases; moreover, the authors also proved that um-PEA reduced the release of IL-6, IL-1β, TNF-α, and IL-18 and inhibited NF-κB activation and the p38/MAPK pathway [63]. The efficacy of adelmidrol (at the dose of 10 mg/kg given for 21 days) in mice with bleomycin-induced pulmonary fibrosis was also investigated by Fusco et al., showing this PEA analogue’s ability to reduce airway infiltration by inflammatory cells and mitigate the production of pro-inflammatory cytokines. Moreover, adelmidrol treatment was able to control the increased oxidant burden and to modulate the JAK2/STAT3 and IκBα/NF-κB pathways [64].

Few data have assessed PEA anti-angiogenic activity in chronic inflammatory conditions. Sarnelli et al. showed that PEA improves inflammation-driven angiogenesis in mice affected by dextran sulphate sodium-induced colitis, reducing the local intestinal damage and affecting disease progression towards carcinogenesis. In particular, the inhibition of colitis-related angiogenesis specifically decreased the release of vascular endothelial growth factor via the PEA-mediated mTOR/Akt axis [65]. PEA also has a role in the gastrointestinal health promoted by interactions with the gut microbiome, and indeed, in vitro addition of endocannabinoids to gut microbiome-like culture may significantly increase the anchoring of beneficial components of the microbiota [66]. A subverted endocannabinoid activity has also been shown in obesity and metabolic syndrome, revealing differences between insulin-resistant postmenopausal obese women and their insulin-sensitive counterparts [67]. Annunziata et al. investigated PEA metabolic activities in the liver of a mouse model of diet-induced obesity, showing its power to normalize glucose and lipid levels via upregulation of GLUT2 protein expression. In particular, PEA decreased high-fat diet-related adiposity and energy expenditure in obese mice, also favoring a shift towards lipid oxidation, which was found to lessen hepatic steatosis progression: the effects of PEA were blunted by AMP-activated protein kinase inhibition, evidencing the involvement of this enzyme in PEA-modulated regulation of lipid metabolism [68]. PEA administration has also been shown to reduce fibrogenesis in a rat model of carbon tetrachloride-induced liver fibrosis via inactivation of stellate and Küpffer cells, hypothesizing that PEA may have a potential place in the management of liver fibrosis [69]. In addition, PEA supplementation has been proven to significantly attenuate renal dysfunction via the NF-κB signaling pathway in a mouse model of renal injury, reducing the biosynthesis of pro-inflammatory molecules and increasing the release of antioxidants [70]. PEA may also significantly downgrade the burden of different pro-inflammatory mediators such as TNF-α, IL-1β, leukotriene B4, prostaglandin E2, and matrix metalloproteinases in patients with osteoarthritis, providing relief in terms of joint swelling and decreasing the risk of irreversible cartilage damage [71].

The potential anti-neoplastic effect of PEA requires further validation in pre-clinical animal models and clinical trials [72]. However, due to a minimal risk of drug–drug interactions and due to the absence of potentially adverse effects, PEA may be useful as a core tool in the palliation of complex patients and in the management of autoinflammatory disorders of childhood, characterized by recurrent episodes of organ-specific inflammation affecting the skin, joints, gut, or central nervous system, without any evidence of autoantibody production or underlying infections, through a specific involvement of the endocannabinoid system, particularly in patients for whom conventional therapies have proven insufficient or intolerable, and mostly without modifying the risk of gastrointestinal bleeding, cardiovascular complications, or renal impairment [73,74].

## 6. Conclusions

PEA is implicated in many physiological and pathological processes with pleiotropic activities and cytoprotective effects, but its pharmacological profile shows to be multi-faceted beyond the well-known anti-inflammatory properties related to microglia and mast cell downregulation, as it also activates the endocannabinoid pathway, displays modulating energy balance, and refines endogenous antioxidant capacity.

Unfortunately, the current evidence about PEA is primarily based on disparate preclinical studies or small cohort trials with many potential biases and lacking randomization or sufficient control groups, which jeopardizes the overall generalizability of their outcomes. Additionally, most available studies differ in the PEA dose used, hampering the resulting conclusive remarks. PEA improved bioavailability, with better efficacy of the micronized formulations, confirms its ultimate potential in different clinical applications, significantly relieving inflammation and also reducing the pro-inflammatory burden of neuropathies or non-neurologic afflictions.

This review has underscored some relevant gaps that may guide future studies exploring PEA efficacy in treating, aside from chronic inflammation and pain control, many human diseases. Clearly, further studies should include larger numbers of patients across diverse populations, and standardized protocols should be used to investigate the exact therapeutic role of PEA, requiring a critical examination of its specific tissue activities and dynamic interaction with endogenous cells in inflammatory diseases.

## Figures and Tables

**Table 1:** List of clinical trials in progress to assess palmitoylethanolamide (PEA) effectiveness as a symptom reliever in various clinical conditions.

Study Number	Trial Description	Study Design	Type of the Study	Status	Referral Center
NCT06273462	To evaluate if the supplementation of PEA (600 mg twice/day) is effective in all-aged patients with chronic pain	Prospective randomized double-blind placebo-controlled	Interventional, phase 2	Recruiting	Navy Medical Center, San Diego, USA
NCT01491191	To evaluate the supplementation of PEA in the prevention of postsurgical pain incidence for patients undergoing urologic and gynecologic surgery	Prospective randomized	Interventional	Recruiting	University of Modena and Reggio Emilia, Italy
NCT05867693	To evaluate both efficacy and safety of co-micronized PEA/polydatin in pediatric patients (>10 years) with irritable bowel syndrome	Prospective randomized double-blind placebo-controlled	Interventional	Recruiting	University La Sapienza, Rome, Italy
NCT06777680	To evaluate the efficacy of PEA and luteolin on early functional recovery in acute ischemic stroke patients treated with thrombectomy	Pilot prospective randomized placebo-controlled	Interventional	Not yet recruiting	Ospedali Riuniti, Trieste, Italy
NCT06063369	To evaluate PEA efficacy in patients with major depressive disorder	Randomized double-blind placebo-controlled	Interventional, phase 2	Recruiting	University of Medicine and Pharmacy, Chisinau, Moldova
NCT05877781	To evaluate dietary supplementation of PEA (400 mg × 3/day) in patients with functional dyspepsia	Randomized double-blind placebo-controlled	Interventional	Recruiting	Universitaire Ziekenhuizen, Leuven, Belgium

## Data Availability

Not applicable.

## Abbreviations

The following abbreviations have been used in this manuscript:

PEA	Palmitoylethanolamide
IL	Interleukin
TNF	Tumor necrosis factor
TNFR	TNF receptor
CCL	Chemochines ligand
PPAR	Peroxisome proliferator-activated receptor
NF-κB	Nuclear factor kappa-light-chain-enhancer of the activated B-cell
MAPK	Mitogen-activated protein kinase
AEA	Anandamide
OEA	Oleoylethanolamide
um-PEA	ultramicronized-palmitoylethanolamide
IFN	Interferon
ASD	Autism-spectrum disorder
ALI	Acute lung injury
